# Sequential Monte Carlo-guided ensemble tracking

**DOI:** 10.1371/journal.pone.0173297

**Published:** 2017-04-11

**Authors:** Yuru Wang, Qiaoyuan Liu, Longkui Jiang, Minghao Yin, Shengsheng Wang

**Affiliations:** 1 Computer Science and Information Technology, North-East Normal University, Changchun, Jilin Province, China; 2 School of Information Engineering, Jilin Business and Technology College, Changchun, Jilin Province, China; 3 Jilin University, Key Laboratory of Symbolic Computation and Knowledge Engineering of Ministry of Education, Changchun, Jilin Province, China; Tianjin University, CHINA

## Abstract

A great deal of robustness is allowed when visual tracking is considered as a classification problem. This paper combines a finite number of weak classifiers in a SMC framework as a strong classifier. The time-varying ensemble parameters (confidence of weak classifiers) are regarded as sequential arriving states and their posterior distribution is estimated in a Bayesian manner. Therefore, both the adaptiveness and stability are kept for the ensemble classification in handling scene changes and target deformation. Moreover, to increase the tracking accuracy, weak classifiers including Support Vector Machine (SVM) and Large Margin Distribution Machine (LDM) are combined as a hybrid strong one, with adaptiveness to the sample scales. Comprehensive experiments are performed on benchmark videos with various tracking challenges, and the proposed method is demonstrated to be better than or comparable to the state-of-the-art trackers.

## Introduction

The goal of visual tracking is to locate the target states over a video sequence. Usually, the tracking-by-detection methods can be categorized into two main branches: the generative model and the discriminative model. This paper focuses on the discriminative model. It considers the tracking problem as a binary classification problem, and the key issue is classification learning.

For sequential arriving video frames, the appearance of target always undergoes great changes caused by complex tracking conditions. In many practical applications [1–4], different types of challenges occur frequently, including illumination variation, target deformation, frequent occlusion and fast motion. Represented by an appropriate visual model, the detection region is projected into the feature space as samples, specifically, positive ones for the target and negative ones for the background. The task of the discriminative model [5, 6] is to learn a classifier capable of distinguishing the positive samples from the negative ones. Both the tracking accuracy and generality ability are significant for an excellent classifier, i.e. the accuracy in building a hyper-plane classifying the positive and negative samples and the generalization ability in assigning the new arriving samples into the right class.

Advidan, for the first time, introduced the classification method for the tracking problem. In 2004, he proposed to solve the tracking problem by employing the popular support vector machine, and proposed Support Vector Tracker (SVT) [7]. It shows promising performance and has received much attention. Similar classifier also includes weakly supervised classifier [8]. In 2007, his other paper, entitled “Ensemble Tracking” [9], was published in PAMI. As the experimental results show, his work revealed exciting robustness and accuracy. The success relies heavily on the excellent ensemble classifiers proposed. It learns a hyper-plane by using a strong classifier combined with several weak classifiers. Since then, the idea of adaboost was introduced into the ensemble tracking method, many methods based on the ensemble idea have been proposed. In 2006, Grabner [10] employed an on-line Adaboost to select features and provided them to particular classifiers. In 2007, Tian [11] proposed an on-line ensemble SVM tracker, additionally in 2010, Thomas [12] proposed a Modular Ensemble Tracker (MET). In 2014, Zhou [13] proposed a dynamic selection strategy with ensemble classifiers.

As a key the ensemble of classifiers provides the tracker with more robustness. Typically, each classifier is assigned to a particular visual cue, and all the classifiers are weighted-combined according to their confidences. As the tracking proceeds, influenced by the tracking conditions and target deformation, the target always shows a variable appearance. Because the confidence of each weak classifier varies with the discriminative ability of the visual cues, they should be updated to maintain sufficient generality of the tracker. The model updating scheme for tracking-by-detection has been widely studied in the literature. There also exist some methods related to the discriminative model. Grabner [10] used Boosting as a feature selector and realized an online boosting tracker. Saffari et al. [14] proposed an online multi-class boosting model. In another approach [15], Random Forests undergoed an online update to grow and discarded decision trees during tracking. Usually, the existing methods adapt the ensemble weights of weak classifiers according to their outputs. However, the posterior distribution of a target is unknown, thus this updating scheme becomes unreliable. Moreover, updating is performed based on the current observation; as a result, the information is in a time sequence. In 2013, Bai et. al. [16]proposed a Randomized Ensemble Tracker, he modelled the ensemble weights by drawing a Dirichlet distribution and updated them randomly. Though good performance was achieved on several challenging tasks, the essential parameters in Dirichlet were still updated by the tracking confidence, which was unreliable. Due to the posterior distribution of estimated ensemble parameters being unknown, the best way is to estimate their states in a Bayesian framework.

Another crucial factor related to tracking performance is the classifier employed. In the existing ensemble trackers, the employed classifiers including SVM [7], fuzzy C-means [17], v-Support vector [18, 19] and Structural Minimax Probability Machine [20] et al. The classifiers are not required to be of strong discriminative ability, because the tracking results are obtained by their combination. Therefore in the designed of classifier, both the samples and the classifier together determine the tracking performance. Accordingly, despite of the less requirement on classifiers, in this paper we made further research on these two issues. It is well known that SVM can be viewed as a learning approach that tries to maximize over the training examples the minimum margin. Reyzin [21] conjectured that the margin distribution rather than the minimum margin, is more crucial to the generalization performance. It has also been disclosed by Gao and Zhou [22] that rather than simply considering a single-point margin, both the margin mean and variance are important. In 2014, Gao and Zhou [23] introduced samples’ distribution data into the definition of the objective function and proposed LDM. Having been tested on many typical datasets, its superiority to SVM was demonstrated. However, the learning step of LDM costs much more time than that of SVM, so employing LDM as the weak classifier will lead to low efficiency. However, it could perform with better generality and accuracy than SVM for the problems that are hard to classify. Therefore, we employ a hybrid classifier combined both SVM and LDM, which will not only ensure the efficiency but also improve the tracking accuracy at the same time.

In this paper, we conduct major research on increasing the discriminating and generalization abilities of an ensemble tracking. Specifically, the SMC method is employed to model ensemble parameters updating. The vector of weights assigned to weak classifiers is viewed as a sequential arriving state in a Bayesian framework. Based on the observation of some random samples, the states of ensemble parameters are approximated by using the Monte Carlo method in the time sequence. Moreover, the sample observation is associated with the outputs of the weak and strong classifiers. To obtain more robustness and generality, this paper generalized LDM to the tracking problem and constructed a hybrid classifier adapted with feature scales. The proposed method is field-tested on benchmark videos including types of challenges. Comprehensive experiments and analysis are performed, its performance is demonstrated to be better than or at least comparable to previous algorithms in the literature.

The rest of this paper is organized as follows: the framework of the proposed SMC-guided ensemble tracker is introduced in Section 2. A pyramid patches-based visual model is stated as the input of ensemble classifiers in Section 3. Then, the employed hybrid weak classifiers are discussed in Section 4. Finally, Section 5 presents the experimental results and analysis to illustrate how the method adapts to various tracking challenges.

## Sequential Monte Carlo-guided ensemble tracking

**Fig 1:**
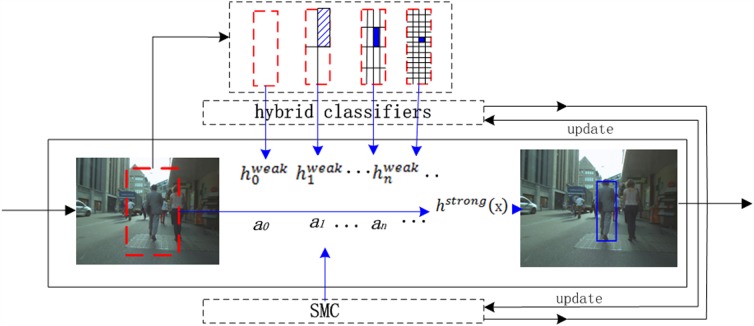
Overview of the proposed method.

Fig 1 gives an overview of the proposed tracking system. The tracking problem is formulated as a binary classification task. At each time step, given the prior outputs from the pre-frame, the tracker detects the target state depending on a strong classifier. The detection region is represented in a multi-scale pyramid model. Scale-adaptive hybrid weak classifiers are weight-combined as the strong classifier. Specially, their weights are modelled in a SMC framework to realize more generality, and both the classifiers group and their weights are updated in the time sequence. In this section, we briefly review the ensemble tracking algorithm and resolve the ensemble updating problem in a SMC framework. Rather than updating the ensemble parameters under the current observation, we represent them as a sequential estimated state and realize updating in a SMC framework.

### Ensemble tracker

Given a set of N weak classifiers, a strong classifier is computed via a linear combination.

$$h^{strong}\left(x\right)=\sum_{n=1}^{N}\alpha_{n}\cdot h_{n}^{weak}\left(x\right)$$ (1)

*α*_*n*_ in this equation is a weight, measuring the confidence of each classifier. For each video frame, we denote the weights of all the weak hypotheses as a vector *V*. The hypothesis $h_{n}^{weak}$ generated by a weak classifier corresponds to a feature and is obtained by applying a defined learning algorithm. In the video sequence, we denote the series of sequentially arriving data sets as *f*_0_, *f*_1_, …, where *f*_*i*_ in our application is the detection windows in the *i*-*th* frame. Represented in a discriminate visual model, a set of features are extracted from *f*_*i*_ as data assigned to the weak classifiers. At time t, given input data **x** ∈ *f_t_*, the task of the tracker is to predict its label *y* ∈ {+1, −1}.

$$y=\left\{\begin{array}{cc} +1, & h^{strong}\left(x\right)\geq \tau \\ -1, & otherwise \end{array}\right.$$ (2)

where threshold *τ* is a model parameter. Then, the target state is estimated by maximizing the confidence map based on all the samples’ outputs.

### Sequential Monte Carlo based tracker updating

Because the tracking conditions undergo time-varying changes, the tracker should also have adaptiveness and generality. We propose updating the ensemble tracker in a Bayesian framework. Specifically, we update both the weight vectors and the pool of weak classifiers after the classification stage in each time step to evolve the model.

The weight vector *V*_*t*_ is adapted at each time step to keep the tracker adaptive. Therefore, the sequentially arriving flow of *V*_*t*_ over the whole video sequence is seen as a state-evolving procedure. Then, from a Bayesian point of view, its updating is estimated by obtaining its posterior distribution. The SMC method provides a good solution to this problem.

Given a sequence *V*_*s*_(*V*_1_, *V*_2_, …, *V*_*m*_) and a corresponding sequence of observations *Z* = (*z*_1_, *z*_2_, …*z*_*m*_), the updating goal is to find those *V*_*s*_(*V*_1_, *V*_2_, …*V*_*m*_) that maximize the posterior distribution

$$P\left(V_{1=\upsilon_{1}},\ldots \;,V_{m=\upsilon m}\right)=p\left(\upsilon_{1:m}|Z\right)$$ (3)

where $\upsilon_{1:m}=\left(\upsilon_{1},\ldots ,\upsilon_{m}\right)\in V_{m}$ is a state space vector representing the possible values of the *V*_*s*_. For each state *υ*_*t*_, there is a corresponding observation *z*_*t*_ for *t* = 1…*m*. Based on the prior probability distribution and current observations, the goal of the weight vector estimation is to find the value ${\hat{\upsilon }}_{t}$ of states *υ*_*t*_ such that

$${\hat{\upsilon }}_{1:m}=argmax_{\upsilon_{1}:m}\left(\upsilon_{1:m}|Z\right)$$ (4)

The Monte Carlo method provides a possible solution to the above equation. Particle Filter (PF) is a recursive Bayesian filter that belongs to the SMC methods. The classical PF framework has been developed for sequential state estimation e.g. tracking [24] [25]. According to the classical PF, at time *t* − 1, the posterior probability distribution *p*(*υ*_*t* − 1_|*Z*_*t* − 1_) is usually approximated by using a finite number (N) of weighted $w_{t-1}^{i}$ samples $p\left(\upsilon_{t-1}|Z_{t-1}\right)\approx {\left\{w_{t-1}^{i},\upsilon_{t-1}^{i}\right\}}_{i=1}^{N}$. Then, the posterior distribution *p*(*υ*_*t*_|*Z*_*t*_) can be approximated using by some weighted samples as

$$p\left(\upsilon_{t}|Z_{t}\right)\approx cp\left(Z_{t}|\upsilon_{t}\right)\sum_{i=1}^{N}w_{t-1}^{i}p\left(\upsilon_{t-1}^{i}|Z_{t-1}^{i}\right)$$ (5)

Because it is difficult to draw samples from the posterior distribution, the importance sampling method is usually performed by a proposal distribution. Samples are drawn from a proposal density *q* as

$$\upsilon_{t}^{i}∼q\left(\upsilon_{t}^{i}\right)≜\sum_{i}w_{t-1}^{i}p\left(\upsilon_{t}^{i}|\upsilon_{t-1}^{i}\right).$$ (6)

The sample weight is usually recursively updated as

$$w_{t}^{i}=p\left(Z_{t-1}^{i}|\upsilon_{t-1}^{i}\right)w_{t-1}^{i}$$ (7)

By updating the ensemble parameters in a PF framework, the ensemble tracker will be adaptive as the tracking continues to realize stability. The weight of each weak classifier is updated not only based on the observation at the current frame but also based on the consistency of the adjacent frames. In such a way, abrupt changes will be avoided and reliable updating is realized.

## Pyramid patches based visual model

Features play an important role in tracking performance [26].We employ a patches-based visual model to generate weak hypotheses. Typically, the existing methods usually employ or construct various independent features as samples and assign them to the corresponding weak classifiers. We represent the detection region in a pyramid model as in [27] and provide features with various scales to the weak classifiers. In this way, the target is observed in a multi-scale model. This model will provide the tracker with much more robustness against occlusion and target deformation.

As shown in Fig 1, at time *t*, the detection region *f*_*t*_ is represented as a multi-scale patches model in a pyramid with four levels. In detail, at the first level, we divide *f*_*t*_ into patches of size *n* × *n* uniformly. At the second and third levels, larger patches that cover different portions of the object are also selected by dividing *f*_*t*_ into the number of 4 × 4, 2 × 2 evenly spaced regions, respectively. At the highest level, it is considered as one patch. For each patch, we extract its 64-bins HSV color/gray-scale histogram and standard histogram of gradients (HOG) [28] features.

For this pyramid model, the extracted samples are in different scales. Therefore, a hybrid weak classifier group is constructed for adaptiveness in the next section.

## Hybrid weak classifiers

As a popular classifier for the existing ensemble trackers, SVM is also employed in our model. It is well known that SVM can be viewed as a learning approach that tries to maximize over training examples the minimum margin. It was disclosed by Gao and Zhou [22] that rather than simply considering a single-point margin, both the margin mean and variance are important. In 2014, Gao and Zhou [23] introduced samples’ distribution data into the objective function and proposed the Large Margin Distribution Machine (LDM). However, it is too strong to be employed as the weak classifier.

Because the target is represented in a multi-scale visual model, each patch corresponds to a particular classifier. Patches in different scales show different discriminate ability. Specifically for a large-scale patch, it is easier to lean a good hyper-plane because its feature vector is usually of large difference. However, regarding to small-scale patches, they always have similar appearance and are difficult to be separated in the feature space. For this type of patches, if the accuracy and generality of the corresponding weak classifiers are insufficient, the robustness and accuracy will be influenced heavily. In this paper, LDM is applied to the small-scale samples. In this way, the performance of the strong classifier will be increased and the computational complexity can be guaranteed.

We set the instance space as $X\in R^{d}$ and the label set as Y={+1,-1}. Regarding classical SVM, its objective function is:

$$\begin{array}{cc} & \min_{\omega ,\xi }\frac{1}{2}\omega^{T}\omega +C\sum_{i=1}^{m}\xi_{i}\\ & s.t.\;y_{i}\omega^{T}\phi \left(x_{i}\right)\geq 1-\xi_{i}\\ & \xi_{i}\geq 0,i=1,\ldots ,m \end{array}$$ (8)

where *ξ* = [*ξ*_1_, …, *ξ*_*m*_]^*T*^ measures the losses of instances and *C* is a trading-off parameter. $\phi (x_{i})$ is a feature mapping of **x** induced by a kernel *κ*.

For LDM, the first- and second-order statistics, that is, the mean and the variance of the margin, are introduced into the learning step. The margin mean is

$$\bar{\gamma }=\frac{1}{m}\sum_{i=1}^{m}y_{i}\omega^{T}\phi \left(x_{i}\right)=\frac{1}{m}{\left(Xy\right)}^{T}\omega$$ (9)

The margin variance is

$$\hat{\gamma }=\frac{1}{m^{2}}\sum_{i=1}^{m}\sum_{j=1}^{m}{\left(y_{i}\omega^{T}\phi \left(x_{i}\right)-y_{j}\omega^{T}\phi \left(x_{j}\right)\right)}^{2}$$ (10)

LDM attempts to maximize the margin mean and minimize the margin variance simultaneously. The soft-margin LDM leads to

$$\begin{array}{cc} \min_{\omega ,\xi }\frac{1}{2}\omega^{T} & \omega +\lambda_{1}\bar{\gamma }-\lambda_{2}\hat{\gamma }+C\sum_{i=1}^{m}\xi_{i}\\ & s.t.y_{i}\omega^{T}\phi \left(x_{i}\right)\geq 1-\xi_{i}\\ & \xi_{i}\geq 0,i=1,\ldots ,m \end{array}$$ (11)

To resolve this constraint optimization problem, the dual coordinate descent method (CD) is employed; and the solution details can be found in the paper [23] of Gao and Zhou.

## Experiments and analysis

We implement the proposed method using MATLAB 2013B and carry out the experiments on a computer with a 3.60 GHz CPU and 8 GB of main memory. The code is realized in Matlab and C. We compare our method with nine state-of-the-art tracking methods on eighteen representative video sequences. All the video sequences are downloaded from the Visual Tracker Benchmark [29]. All the quantitative comparison metrics in this paper are based on two widely used comparison metrics: ACLE(Average Center Location Errors) [30] and AOR(Average Overlap Ratio) [31].

### Parameter selection

At each time step, to estimate the ensemble parameters, a particles set is generated based on a Gaussian Perturbation to the prior state. In this paper, we generate 500 samples for a particle set. Specifically, given a prior particle $V_{t-1}^{i}$, a subsequent particle $V_{t}^{i}$ is generated as

$$V_{t}^{i}=V_{t-1}^{i}+G\left(\mu ,\sigma^{2}\right)$$ (12)

where *μ* and *σ* are the mean and variance values of the Gaussian distribution, respectively. Specially, *μ*=0 and *σ* is set by another Gaussian Perturbation. In tracking process, the classifier pool and the weight of each weak classifier varies with time, the Gaussian should not be a fixed value, so we designed the second Gaussian Perturbation as mean value set to be the minimum weight and variance set to be 1/N (N is the number of classifiers). To update the weight *w*^*i*^ of each particle, its observation $p(Z_{t-1}^{i}|\upsilon_{t-1}^{i})$ in Eq (7) is obtained based on evaluating the performance as

$$p\left(Z_{t-1}^{i}|\upsilon_{t-1}^{i}\right)=\frac{2}{1+e^{-s_{i}w_{i}}},$$ (13)

where *s*_*i*_ and *w*_*i*_ denote the sign and weight, respectively. Their values are determined by comparing the output of the weak classifier with the overall output. If $h_{n}^{weak}$ correctly recognizes the target object, then *s*_*i*_ is set to be 1; otherwise, it is set to be -1. The weight *w*_*i*_ is set to be the classification margin.

For the LDM classifiers, the radial bias kernel function is employed, with *γ* set as 2∼10. For the SVM classifiers, the linear kernel function is employed, with the tolerance of termination criterion e set as 0.1.

### Quantitative comparison

**Fig 2:**
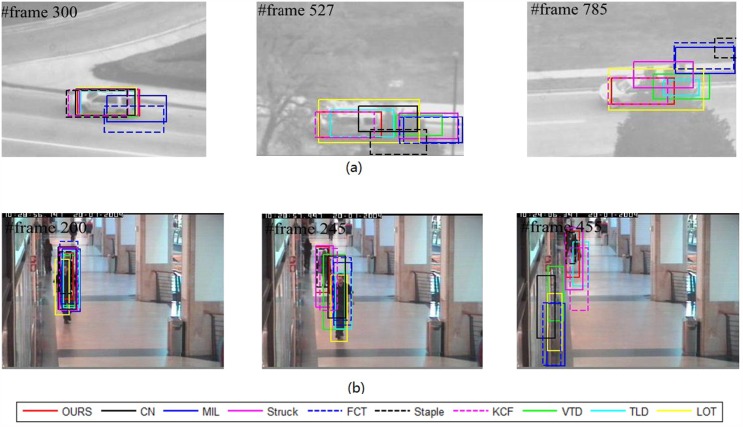
Tracking results for videos with challenges of “partial occlusion”: (a) “Suv” and (b) “Walking2”.

**Fig 3:**
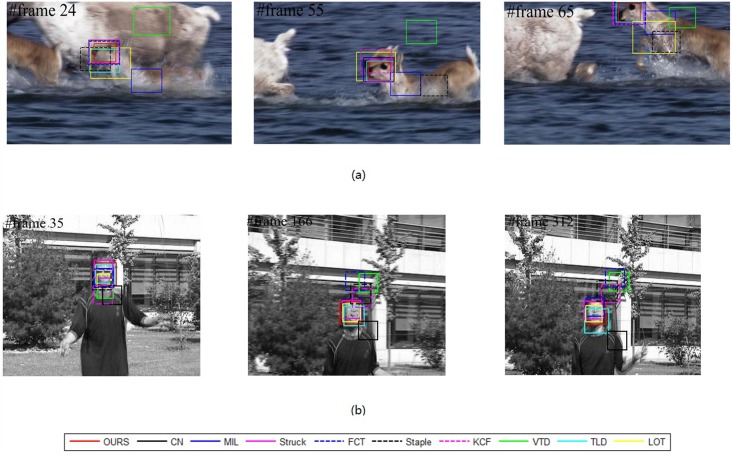
Tracking results for videos with “abrupt motion”: (a) “Deer” and (b) “Jumping”.

**Fig 4:**
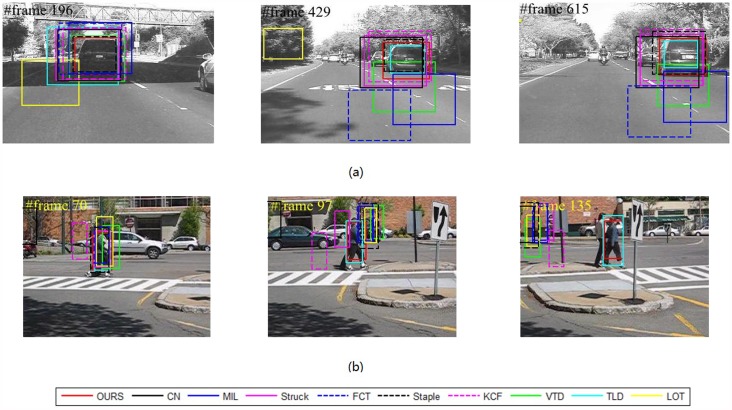
Representative tracking results for videos with “variable illumination”,i.e.,(a) “Car4” and “complex background”,i.e.,(b) “Couple”.

Our method is compared with nine outstanding state-of-the-art tracking methods, including “the visual tracking decomposition” (VTD) [32], “the multiple Instance Learning” (MIL) [33], “the tracking learning detection” (TLD) [34], “structured output tracking” (Struck) [35], “locally orderless tracking” (LOT) [36], “Adaptive color attributes for real-time visual tracking” (CN) [37], “Fast Compressive Tracking” (FCT) [38], “High-Speed Tracking with Kernelized Correlation Filters” (KCF) [39] and Complementary Learners for Real-Time Tracking (Staple) [40]. The tracking results for some key frames of eight representative video sequences are reported, as shown in Figs 2–4, respectively.

**Fig 5:**
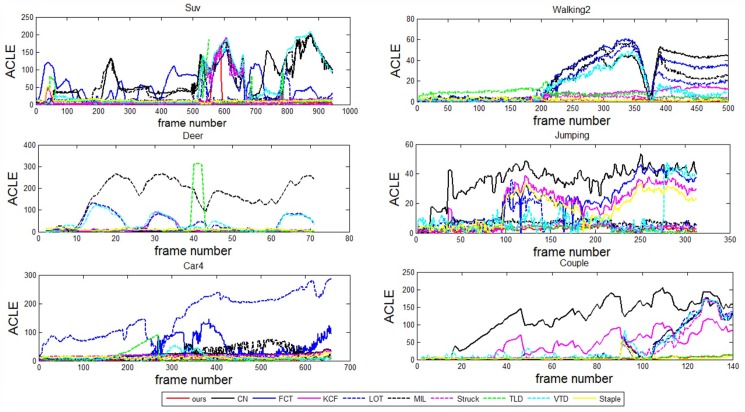
ACLE comparison curves for eight representative challenging video sequences.

**Fig 6:**
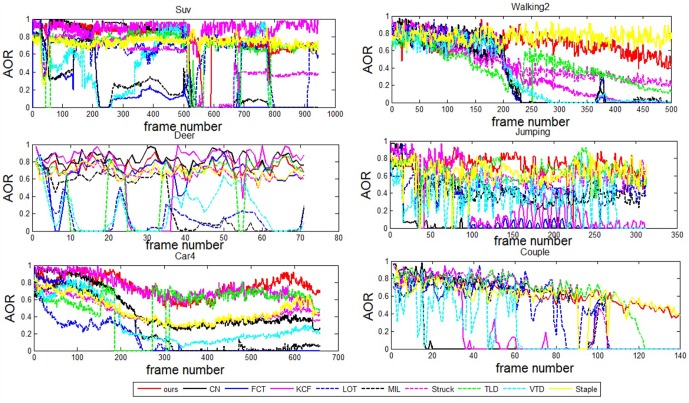
Comparison curves of AOR for eight representative challenging video sequences.

Their quantitative comparisons are labelled in Tables 1 and 2 with respect to the metrics of ACLE and AOR. Most of their ACLE and AOR curves are presented in Figs 5 and 6 over the whole sequence. Overall, our method performs with better or at least comparable performance in comparison with the state-of-the-art methods. These results are analyzed in detail in the following section.

**Table 1:** Statistical comparison of ACLE for eighteen video sequences, where the bold fonts indicate those with the best performance and the underlined values indicate the second-best ones.

	VTD	MIL	TLD	Struck	LOT	CN	FCT	KCF	Staple	ours
BOY	7.17	6.37	3.79	2.97	42.1	4.77	6.07	1.97	1.92	**1.32**
Basketball	16.87	95.13	-	103.3	54.04	9.45	77.4	**5.02**	11.44	11.73
Car4	19.26	30.37	53.14	13.3	144.86	18.54	37.7	9.58	12.1	**2.67**
Coke	44.71	25.78	23.3	**7.89**	44.38	30.9	12	12.67	16.8	13.67
Couple	65.94	31.24	4.42	16.39	30.59	118	31.9	45	4.3	**2.98**
Deer	177.63	55.81	-	5.89	47.32	**2.8**	4.4	11.6	10.5	3.02
Dog1	12.76	7.16	4.53	5.5	13.77	3.45	5.71	**2.87**	6.2	8.56
FaceOcc1	9.25	17.2	14.78	7.54	19.59	12.9	21.9	5.9	15.3	**5.37**
FaceOcc2	5.01	7.87	8.47	16.96	13.18	19.9	4.01	6.03	8.3	**3.34**
Fish	8.82	25.63	8.31	14.56	32.77	35.7	8.49	2.91	8.6	**2.6**
Football1	5.44	4.87	4.87	11.86	3.37		16.4	3.25	**2.3**	4.06
Girl	6.19	11.78	5.94	**2.66**	20.07	12.5	12.96	8.39	7.9	7.98
Jumping	27.07	12.7	**2.24**	3.29	15.65	35.5	22	19.34	17.3	2.35
Suv	64.87	71.6	-	36.45	25.22	123	73.8	**2.92**	11.2	8.87
Sylvester	14.81	9.39	8.09	**4.63**	12.17	9.46	6.55	8.57	10.6	5.06
Tiger2	40.91	30.05	29	22.6	68.66	18.3	12.4	44.22	10.5	**4.9**
Walking	6.8	1.87	67.1	1.57	1.96	7.54	1.85	2.71	2.5	**1.43**
Walking2	13.84	20.02	7.02	3.16	19.87	21.34	23.5	6.66	1.8	**1.49**
average	30.4	25.8	16.3	15.6	33.9	32.1	21.1	11.1	8.5	**5.08**

**Table 2:** Statistical comparison of AOR for video sequences, where the bold fonts indicate those with the best performance and the underlined values indicate the second-best ones.

	TLD	LOT	MIL	Struck	VTD	CN	FCT	KCF	Staple	ours
BOY	0.69	0.47	0.51	0.68	0.6	0.61	0.63	0.65	**0.78**	0.72
Basketball	0.06	0.46	0.22	0.18	0.63	0.64	0.23	0.68	**0.69**	0.56
Car4	0.33	0.03	0.23	0.44	0.35	0.49	0.24	0.73	**0.79**	0.75
Coke	0.33	0.01	0.24	**0.58**	0.13	0.3	0.36	0.39	0.58	0.53
Couple	0.61	0.45	0.48	0.53	0.2	0.1	0.48	0.22	0.5	**0.63**
Deer	0.5	0.16	0.34	0.68	0.07	**0.83**	0.67	0.71	0.68	0.75
Dog1	0.58	0.58	0.53	0.54	0.54	0.45	0.48	0.59	**0.77**	0.51
FaceOcc1	0.53	0.4	0.57	0.68	0.64	0.58	0.55	0.62	0.7	**0.77**
FaceOcc2	0.57	0.45	0.6	0.68	0.68	0.53	0.65	0.63	**0.73**	0.72
Fish	0.59	0.24	0.38	0.55	0.62	0.3	0.67	0.59	0.69	**0.88**
Football1	0.36	0.61	0.6	0.46	0.54	-	0.17	0.48	**0.76**	0.58
Girl	0.56	0.36	0.39	**0.68**	0.58	0.42	0.36	0.51	0.55	0.53
Jumping	0.66	0.46	0.4	0.64	0.22	0.1	0.2	0.29	0.24	**0.7**
Suv	0.68	0.57	0.21	0.5	0.34	0.1	0.49	**0.88**	0.7	0.73
Sylvester	0.57	0.48	0.54	0.65	0.53	0.64	**0.68**	0.66	0.59	0.63
Tiger2	0.21	0.13	0.41	0.45	0.28	0.57	0.39	0.35	0.56	**0.69**
Walking	0.33	0.66	0.51	0.55	0.55	0.57	0.53	0.71	**0.69**	0.67
Walking2	0.41	0.31	0.29	0.47	0.32	0.35	0.28	0.38	**0.76**	0.69
average	0.48	0.38	0.41	0.55	0.43	0.44	0.43	0.54	0.65	**0.67**

### Tracking result

The test sequences are categorized into four typical challenging problems: “partial occlusion”, “abrupt motion”, “illumination variation” and “complex background”.

#### Partial occlusion

The targets in sequences “Suv” and “Walking2” suffer heavy or longtime occlusion. In the Suv sequence, Fig 2 shows that our method performs a accurate tracking in terms of position and scale when the targets undergo severe occlusion and deformation. For example, at frames #527 and #785 of Fig 2(a), the other methods lose the target. For the sequence “Walking2”, our method, Struck and Staple perform better than the other methods at frames #200, #245, #455. In comparison, the other methods suffer from sever drift and some of them completely fail. Furthermore, both the ACLE and AOR curves and the statistical values show that our method performs with better accuracy than Struck and Staple. This dominance can also be observed from the tracking results at frames #245 and #455. The success is attributed to the facts that: (1) the detection region is represented as pyramid patches, both the global and local features are extracted, and this visual model provides the tracker with robustness against occlusion; (2) hybrid classifiers are combined with adaptiveness to scales, which enhances the discriminate ability to a large extent; (3) the ensemble parameters are modeled in a SMC framework, and this method assigns the ensemble framework with stability and generality.

#### Abrupt motion

It is a challenging task when the target moves with abrupt motion because the images may be blurred. As shown in Fig 3(a), over the whole sequence, a deer jumps up and down so fast and the water around occludes its face occasionally. Particularly, in frame #24, the deer jumps down abruptly, which causes the video resolution to decrease. This abrupt motion leads to some methods having declining accuracy. Finally, after several abrupt motions, at approximately frame #65, only our method, TLD and Struck survive. In comparison, our method outperforms.

Moreover, our method also succeeds for the “Jumping” sequence, as shown in Fig 3(b), where similar situations challenge the methods. A man jumps severely and the target face also shows scale changes. Particularly, he abruptly changes his direction of movement (up to down or down to up), e.g. at frame #35 and #166. Our method shows excellent robustness against such challenges. The robustness is attributed to the employment of an adaptive ensemble tracker. On one hand, the hybrid classifiers employ different classify methods on patches in different scales to separate target from background. The adaptiveness to scale has been improved. On the other hand, the update strategy for tracking parameters in Sequential Monte Carlo framework can efficiently avoid random changing, which is caused by generating Dirichlet distribution complete randomly. That is also the key factor of our method to be successful in handling abrupt motion.

#### Illumination variation

Illumination is a typical challenge in many applications. The “Car4” sequence is a representative one with large illumination variation. For example, at frame #196 of Fig 4(a), the target car suffers from a heavy shadow. This difficulty influences the accuracy of most methods. Particularly, after frame #196, the accuracies of VTD, FCT, LOT and MIL begin to decline. This situation continues until approximately frame #615; then, drifts become unavoidable for TLD and Struck. Nevertheless, our method realizes a stable tracking along the whole sequence.

The illumination changes impose variations on both the positive and negative samples. Correspondingly, the discriminative abilities of the weak classifiers also vary much. The classifiers assigned to patches with exposure or shadows will show decreased accuracy. In our tracker, these variations are reflected in the proposed SMC framework. Therefore, the tracker will receive much robustness.

#### Complex background

For a binary classification problem, handling the confusion boundary is a challenging task. For a tracking problem, the complex background is also challenging. As shown in Fig 4(b), the companion and the target walker have similar appearance. This kind of difficulty challenges the visual model and the classifiers. From the tracking results and the ACLE and AOR curves, the outstanding performance of our method is obvious.

As shown in Fig 4(b), the targets move in variable scenes. In such cases, the challenges mainly come from the variance of negative samples in the discriminative model. To handle such cases, the classifiers should have sufficient generality. The introduction of LDM to our model provides the tracker with more robustness against these challenges.

## Conclusions

In this paper, we extensively researched the ensemble classification method, with respect to its discriminate and generalization ability, to realize robust and accurate tracking. The detection region is represented as pyramid patches. Correspondingly, some hybrid classifiers are combined in an ensemble way. To increase the generality and stability, the ensemble parameters are considered as a state and modeled in a SMC framework. The field test demonstrates the effectiveness of the proposed method. In our experiments, we find that the representation of each local patch greatly determines the tracking performance. In our future work, we will develop a visual model with more discriminative ability.
